# rspatialdata: a collection of data sources and tutorials on downloading and visualising spatial data using R

**DOI:** 10.12688/f1000research.122764.1

**Published:** 2022-07-11

**Authors:** Paula Moraga, Laurie Baker

**Affiliations:** 1 Computer, Electrical and Mathematical Sciences and Engineering Division, King Abdullah University of Science and Technology (KAUST), Thuwal, 23955-6900, Saudi Arabia; 2 College of the Atlantic, 105 Eden St, Bar Harbor, ME, 04609, USA

**Keywords:** Spatial data, open data, visualization, maps, sustainable development goals, R

## Abstract

Spatial and spatio-temporal data are used in a wide range of fields including environmental, health and social disciplines. Several packages in the statistical software R have been recently developed as clients for various databases to meet the growing demands for easily accessible and reliable spatial data. While documentation on how to use many of these packages exist, there is an increasing need for a one stop repository for tutorials on this information. In this paper, we present *rspatialdata* a website that provides a collection of data sources and tutorials on downloading and visualising spatial data using R. The website includes a wide range of datasets including administrative boundaries of countries, Open Street Map data, population, temperature, vegetation, air pollution, and malaria data. The goal of the website is to equip researchers and communities with the tools to engage in spatial data analysis and visualisation so that they can address important local issues, such as estimating air pollution, quantifying disease burdens, and evaluating and monitoring the United Nation’s sustainable development goals.

## Introduction

Spatial data plays a crucial role in a wide range of disciplines, such as environment, health, agriculture, economy and society, and can help governments, companies and citizens improve decision-making. A key example is the use of spatial data by statistical offices worldwide to improve the evaluation and monitoring of the United Nations’ Sustainable Development Goals (SDGs) including those related to health, poverty, inequality, climate and the environment. ^1^

Spatial data are critical in determining the future of endangered and threatened species, ^2^ assessing current and future air quality ^3^ and its effect on population health, and for revealing health inequalities and the early warning of infectious disease outbreaks. ^4^ For example, mapping and analysis of spatial data are critical in the development of management plans to ensure the efficient use of natural resources such as land and water so that the benefits of these resources can be enjoyed by future generations. ^5^ Many of these issues do not occur in isolation. Tackling the SDGs requires the integration and combination of data from different sources including social, economic and environmental data. Location often provides the link between these otherwise disparate datasets. High-resolution spatial data is crucial to tailoring management plans to local situations.

The way we monitor change is being rapidly transformed by advances in technology, computing, and data science techniques. Spatial and spatio-temporal data are becoming increasingly common due to advances in both data collection and management. Novel open data sources such as satellite imagery, remote sensing, and Global Positioning System (GPS) data can be collected in large quantities at high spatial and temporal resolutions, at relatively low cost. At the same time, administrative spatial data are becoming increasingly available in open formats. These data are obtained by registries, surveys, and monitoring stations as well as through community-contributed data platforms. Despite a wealth of large and diverse spatial data sources, spatial data may still be hard to find, difficult to use, or not readily accessible. These hurdles limit the re-use of data and their potential impact. These challenges have been recognised for all scientific data, including spatial, and have led to the development of the Findable, Accessible, Interoperable and Reusable (FAIR) guiding principles for scientific data management and stewardship. ^6^ To maximise their value, data should be FAIR. The first step in (re)using the data is to find them. Therefore, data files should also include descriptive metadata that makes them easily findable for both humans and computers. Once the data are found, users also need to know how data can be accessed, possibly including authentication and authorisation. Data also needs to be interoperable so they can be integrated with other data and interoperate with applications or workflows for analysis, storage and processing. Finally, data should be reusable and to achieve this, they should be well-described so that they can be used and extended in different settings.

R ^7^ is a powerful language for statistical programming that incorporates a wide range of packages that can be used for data access, manipulation, analysis and visualisation. ^8^^,^^9^ Moreover, R includes several packages that act as clients for various spatial databases and repositories to meet the growing demands for easily accessible and reliable spatial and spatio-temporal data. While documentation and many open source repositories on how to use these packages to access these data sources exist, there is an increasing need for a one stop repository for information about these data sources and tutorials on how to access them using these packages.

Here, we present rspatialdata, a website that presents a collection of reproducible tutorials on how to download, manipulate and visualize a wide range of spatial data including administrative boundaries, population density, climate and health data using the statistical software R. The website makes it easier for individuals to explore, access and use a range of spatial data facilitating the conversion of data into tangible impacts. *rspatialdata* makes these diverse data more Findable and Accessible by grouping instructions together in one place and promoting them to the R community. Interoperability and Reuse are made easier by demonstrating how to read and manipulate the data in a common analysis system with tutorials that promote the reuse of data and analyses.

## Methods

### Implementation

The tutorials presented in *rspatialdata* have been created using the open-source R Project for Statistical Computing (RRID:SCR_001905) ^7^ and a number of R packages that allow us to download spatial data corresponding to specific geographic regions and periods of time, as well as to manipulate and visualize the data. Here, we provide a description on how to install the statistical software R and R packages. Then, we show an example on how to download and visualize one of the datasets presented in the website, namely, maximum temperature data. The complete code for all the tutorials can be found at the *rspatialdata* website, and a summary of the datasets and associated R packages included in the website are summarized in Table 1. The code is available from GitHub and is archived with Zenodo. ^87^

**Table 1.** All the datasets included in the *rspatialdata* website and databases and R packages that can be used to retrieve them.

Data	R package	Database
Administrative boundaries	rgeoboundaries	geoBoundaries
Population	wopr	WorldPop
OpenStreetMap	osmdata	OpenStreetMap (OSM)
Elevation	elevatr	AWS Terrain Tiles
Temperature	raster	WorldClim
Rainfall	nasapower	NASA-POWER Project
Humidity	nasapower	NASA-POWER Project
Vegetation	MODIStsp	Moderate Resolution Imaging Spectroradiometer (MODIS)
Land cover	MODIStsp	Moderate Resolution Imaging Spectroradiometer (MODIS)
Air pollution	openair	UK Department for Environment Food & Rural Affairs
Demographic and Health Surveys (DHS)	rdhs	DHS Program
Malaria	malariaAtlas	Malaria Atlas Project (MAP)
Species Occurrence	spocc	Global Biodiversity Information Facility (GBIF)

### Installation of R and R packages

R ^7^ is a free, open source, software environment for statistical computing and graphics with many useful packages for importing and manipulating data, statistical modeling, and visualization. R can be downloaded and installed from the Comprehensive R Archive Network (CRAN) (RRID:SCR_003005). R packages can be installed from CRAN with the function install.packages() passing the name of the package as first argument in quotes. Then, to use the package, the package needs to be loaded with the function library(). For example, we can install and load the visualization package ggplot2 by typing install.packages("ggplot2") and library(ggplot2).

### Example of a tutorial: Downloading and visualizing temperature data

The WorldClim (RRID:SCR_010244) ^10^ database contains global weather and climate data for historical and future conditions at high spatial resolution. These datasets can be easily downloaded with the R package raster, ^11^ which implements several functions for reading, writing, manipulating, analyzing and modeling of spatial data. To use the raster package, we first need to install it and load it. Then, to download data, we can use the getData() function of the raster package by specifying several arguments about the dataset we wish to obtain. For example, to download global maximum temperature, we specify the database name ( *e.g.*, "worldclim"), the variable we want to download ( *e.g.*, "tmax"), and the spatial resolution in minutes of a degree as follows.

install.packages("raster"); library("raster")
dataset <- getData (name = "worldclim", var = "tmax", res = 10)


The downloaded object contains 12 files that correspond to the maximum temperature observed each month. We can manipulate the downloaded object to obtain temperature values for a specific month or average temperature spanning several months, and use other R packages to model and visualize the data.

library(ggplot2)

gain(tmax_data) <- 0.1 # Convert temperature to degrees Celsius

# Converting the raster object into a dataframe
tmax_data_may_df <- as.data.frame(tmax_data$tmax5, xy = TRUE, na.rm = TRUE)
rownames(tmax_data_may_df) <- c()

ggplot(data = tmax_data_may_df, aes(x = x, y = y)) +
geom_raster(aes(fill = tmax5)) +
labs(
title = "Maximum temperature in May",
subtitle = "For the years 1970-2000"
) +
xlab("Longitude") +
ylab("Latitude") +
scale_fill_gradientn(
name = "Temperature (^°^C)",
colours = c("#0094D1", "#68C1E6", "#FEED99", "#AF3301"),
breaks = c(-20, 0, 20, 40)
)


## Operation

The software R and RStudio are available for Linux, Mac, and Windows operating systems. It is recommended running these tutorials on a recent version of R (at least R version 4.1.1) and RStudio (at least RStudio version 2021.09.0). R can be downloaded from CRAN, the comprehensive R archive network ( https://cran.r-project.org/). CRAN is composed of a set of mirror servers distributed around the world and is used to distribute R and R packages. RStudio is an integrated development environment, or IDE, for R programming. RStudio can be downloaded and installed from http://www.rstudio.com/download. It is recommended updating both R and RStudio at least once a year to keep up to date with the most recent changes.

## Use cases

The *rspatialdata* website provides a collection of data sources and tutorials on how to download and visualize spatial data, including administrative boundaries, population, elevation, climatic variables, and health data. These data come from different sources. For example, remote sensing data are acquired by sensors that are not in contact with the target of investigation and can be done, for example, using satellites orbiting the Earth. Remote sensing is used to measure everything from land cover ( *e.g.*, water, habitat), environmental phenomena ( *e.g.*, elevation, water and sea temperature), to our human footprint ( *e.g.*, night light maps). More precise information on a range of environmental and climatic variables such as temperature, rainfall and air pollution can be obtained using monitoring stations placed at specific places that provide ground measurements of these variables during different periods of time. Surveys are also useful to obtain information about health, economy and social characteristics of the population at the local scale. Here, we describe the data sources included in the website, as well as the R packages that allow us to download the data. We also give examples of where these data can be used to solve problems in different disciplines such as health, ecology and the environment.

### Administrative boundaries

Administrative boundaries are an essential component for making maps and define the spatial extent needed for electoral, planning and statistical studies. These boundaries, which often guide the spatial scale at which data is collected, offer important context to a wide-range of issues. geoBoundaries ^12^ is an open license resource database of political administrative boundaries. The R package rgeoboundaries ^13^ is an R client for the geoBoundaries application programming interface (API) that allows us to download administrative boundaries of countries at different administrative levels.

This package has been used as a visualization tool for the study of many different real-world problems, such as mapping coronavirus-19 presence in Vietnam, ^14^ understanding the impact of Global Environment Facility Projects in Uganda ^15^ and the influence of travel time to health facilities on stillbirths in Nigeria. ^16^

The *rspatialdata* tutorial includes an example of how to retrieve the administrative boundaries of single and multiple countries at different administrative boundary levels. It also covers how to download and visualize these data using the sf ^17^ and leaflet ^18^ packages.

### Population

Knowing population sizes and their spatial distributions is crucial for many critical decisions from improving access to health, transportation and energy, to planning and building more resilient and sustainable cities. WorldPop ^19^ aims to provide an open access archive of spatial demographic datasets with a focus on low and middle income countries (LMICs) to support development, disaster response and health applications.

Population data from WorldPop has been used extensively to map health conditions such as cancer, ^20^ child growth failure, ^21^ HIV prevalence, ^22^ and the burden of cholera ^23^ in Africa. It has also been used to map local variation in educational attainment in Africa, ^24^ to evaluate the reduction of tree cover in West African Woodlands ^25^ and to assess clean air in the context of the SDGs. ^26^

The WorldPop Open Population Repository provides access to high-resolution population estimates for individual countries and these data can be obtained with the R package wopr. ^27^ The *rspatialdata* tutorial shows examples on how to use wopr to download population data for different countries and administrative levels.

### OpenStreetMap (OSM) data

OSM ^28^ is a collaborative project to create a free editable map of the world. OSM is built by a community of mappers that contribute and maintain global data about roads, trails, cafés, railway stations, and more. OSM data can be used in many ways. For example, as a basemap to put other data into context, for routing or navigation, and for planning or logistics for humanitarian groups, utilities and governments. OSM data have been used in a wide range of applications including flood inundation modeling, ^29^ air pollution exposure, ^30^ assessment of socio-economic factors and property prices, ^31^ and for the study of crime and place. ^32^

The package osmdata ^33^ allows us to easily import OSM data in R. The *rspatialdata* tutorial includes an example of how to retrieve OSM data using the osmdata by creating a bounding box and a query and how to visualized the data with ggplot2, ggmap ^34^ and leaflet. ^18^

### Elevation

Elevation data are important in many different applications. For instance, for environmental problems, elevation data have been used as a tool to study the land cover change over the years, in particular, the evolution of European forest cover. ^35^ As another example, researchers also have been using elevation data as a complementary source of information in the analysis of species connectivity through genetic structure. ^36^^,^^37^

For retrieving elevation data from many different regions, one may choose to work with the the elevatr package. ^38^ elevatr provides access to elevation data from several web services including the Amazon Web Services Terrain Tiles, ^39^ the Open Topography Global Datasets API, ^40^ and the USGS Elevation Point Query Service. ^41^

The *rspatialdata* tutorial includes an example of how to retrieve and visualize point elevation data for the USA and raster elevation data from a digital elevation model (DEM) for global elevation data.

### Climate data: temperature and precipitation

WorldClim ^10^ is a database that provides high spatial resolution global weather and climate data for historical and future conditions. For example, it provides monthly climate data for minimum, mean, and maximum temperature, precipitation, solar radiation, wind speed, water vapor pressure, and for total precipitation.

These data may be applicable in many different areas. For environmental problems, it has been used for the study of the global tree restoration potential, ^42^ the understanding of temperature profile in forest regions, ^43^ and the monitoring of drought in South Asia. ^44^ In ecology, to understand geographic distribution of sloths in Costa Rica. ^2^ In health and disease-control related problems, these data have been used, for example, in the study of the levels of arsenic in groundwater, ^45^ the prediction of lymphatic filariasis prevalence in sub-Saharan Africa, ^46^ and the loss of biodiversity on Earth due to the amphibian chytridiomycosis panzootic disease. ^47^

The package raster ^11^ allows us to easily download the WorldClim data as well as to manipulate and analyze spatial datasets. The rspatialdata tutorial includes an example of how to retrieve maximum temperature data from the WorldClim database and visualize the monthly maximum and mean monthly temperature and other bioclimatic variables over time using ggplot2 and the sf package. ^17^

### Rainfall and humidity

The NASA Prediction Of Worldwide Energy Resources (POWER) Project ^48^ provides meteorology, surface solar energy and climatology data for support of renewable energy, building energy efficiency and agricultural needs. Data retrieved from the NASA POWER Project have been used in a few different applications. For example, POWER data have been used in the study of the potential utilization of wind electric pumping systems for water distribution in Cameroon, ^49^ in the analysis of photovoltaic systems usage in China ^50^ and in the study of *Dunaliella salina* (a type of green micro-algae) cultivation. ^51^

nasapower ^52^ aims to make it quick and easy to automate downloading NASA-POWER data in R. In *rspatialdata*, we show how to use this package to download rainfall and humidity.

### Vegetation and land cover

Vegetation data are used in a wide variety of applications ranging from environmental applications, such as the rice crop monitoring in Europe, ^53^ to health and disease-control applications, such as malaria transmission dynamics in an indigenous province in Panama. ^54^

Vegetation data are captured using Moderate Resolution Imaging Spectroradiometer (MODIS), an instrument onboard the Terra and Aqua NASA scientific research satellites. MODIS captures data in 36 spectral bands in three spatial resolutions across the surface of the earth. Data products derived from these observations include features of the atmosphere, land, cryosphere, and ocean, made available at different frequencies and spatial resolutions. Each data product contains multiple product layers, including original MODIS layers, quality layers and spectral indexes, produced at different intervals and at different spatial resolutions. User guides on each of the product areas are available, which provide in-depth explanations on them.

The *rspatialdata* tutorial shows how to use the R package MODIStsp, ^55^ which acts as a client for downloading time series and raster images derived from MODIS Land Product data. Specifically, it shows how to download MODIS Vegetation Index Products (NDVI and EVI) ^56^ and the MODIS Land Cover Products. ^57^

### Air pollution

Air pollution data can be of interest for many different agents, from the government to the general population. In this sense, many different studies have been conducted regarding how the UK and other countries have been suffering from different types of pollutants—for instance, on how wood-burning has impacted the PM _10_ levels in London, ^58^ or how the level of air pollution has a direct impact on the population’s health, ^3^ or even how people from different socioeconomic groups may be exposed to different levels of air pollution depending on their commute in London. ^59^

*UK Air* is a UK air quality database provided by the Department for Environment Food & Rural Affairs. ^60^ The database provides daily information about the level of pollution for different pollutants ( *e.g.*, ozone, carbon monoxide, PM _2.5_) across the United Kingdom and its territories. Although there are many different ways to retrieve data from this database, one convenient option is using the openair ^61^ R package.

The openair package provides a set of functions to import and work with these datasets, which are documented in the openair's manual. ^62^ The *rspatialdata* tutorial includes an example of how to retrieve and visualize data from a specific monitoring network named Automatic Urban and Rural Network (AURN).

### Demographic and Health Surveys (DHS)

The Demographic and Health Surveys (DHS) Program ^63^ collects, analyzes, and disseminates country-wide subnational level data on population, health, nutrition and HIV. The objective of the DHS Program is to improve and institutionalize the collection and use of data by developing countries for program monitoring and evaluation and for policy making. The R package rdhs ^64^ provides a wrapper to the DHS program API, and can be used to identify particular datasets and download them in R *via* the DHS API. Examples of issues that have been investigated using DHS data include household smoke-exposure risks associated with cooking fuels and cooking places in Tanzania, ^65^ determinants of unmet need for family planning and implications for women’s health in Gambia & Mozambique, ^66^ and household access to improved drinking water sources and toilet facilities in Ethiopia. ^67^

The *rspatialdata* tutorial includes different examples of options on how to retrieve datasets and DHS surveys for an analysis through the DHS API and DHS website from R. And how to search for a specific DHS survey using tag words demonstrating how to extract surveys on Malaria in Rwanda and Tanzania as a case study.

### Malaria

The Malaria Atlas Project (MAP) ^68^ aims to better understand the global landscape of malaria risk, how this is changing, and the impact of malaria interventions to support malaria intervention and eradication efforts. As part of its work, MAP assembles an extensive collection of malaria data, including parasite rate data ( *Plasmodium falciparum* and *Plasmodium vivax*), vector occurrence, and satellite images capturing conditions that influence malaria transmission. malariaAtlas ^69^ is an R package to open-access malaria data hosted by MAP and can be used to download all publicly available parasite rate survey points, mosquito occurrence points and raster surfaces from the MAP servers as well as utility functions for plotting the downloaded data. Data provided by malariaAtlas can be used to explore the spatial and spatio-temporal patterns of malaria risk as well as to feed into spatial models of the risk of malaria. Several studies have used MAP data for different purposes, including mapping the global endemicity and clinical burden malaria, ^70^ understand the associated patterns of insecticide resistance in field populations of malaria vectors across Africa, ^71^ and assess the population coverage of artemisinin-based combination treatment and *Plasmodium falciparum* infection in Africa. ^72^

The *rspatialdata* tutorial includes examples of how to retrieve and visualize malaria data from the malariaAtlas package including parasite rate (PR) survey data, vector occurrence data, and rasters of modelled malaria research outputs.

### Species occurrence

The information of observed species play an import role in ecological studies, which motivates the existence of different repositories containing these type of data. Examples include GBIF - Global Biodiversity Information Facility (RRID:SCR_005904), ^73^ Biodiversity Information Serving Our Nation (BISON), ^74^ eBird, ^75^ and VertNet. ^76^ Most of these repositories allow researchers to retrieve data using different methods. In R, the aforementioned platforms can be accessed through the rgbif, ^77^ rbison, ^78^ rebird, ^79^ and rvertnet ^80^ packages, respectively. However, in order to integrate all these datasets and interact with them using just one tool, one could choose to work with the spocc package. ^81^ As an example, and aiming to model sloths occurrence in Costa Rica, spocc was used to retrieve relevant data from GBIF. ^82^ Other case studies may include modeling migratory movements of birds ^83^ or estimating population size based on species occurrence. ^84^

The *rspatialdata* tutorial includes an example of how to retrieve and visualize species occurrence data by creating a query for a species latin name using the spocc package.

## Discussion

Open and reliable data are crucial for solving global challenges and monitoring the UN Sustainable Development Goals by 2030, including those for improving health, reducing inequalities, and protecting the environment. Accessible spatial data in particular are key to understanding diverse questions ranging from disease spread to climatic trends and necessary for evaluating the impact of interventions and policy decisions.

In this paper, we present *rspatialdata*, a website containing a collection of data sources and tutorials on downloading and visualising spatial data using the statistical software R. The website represents an important step towards helping users find, access and visualize spatial data. As a one-stop repository for tutorials on accessing spatial data, we aim to provide an overview for users on what spatial data is available and how it can be accessed from R. We use motivating examples in the tutorials to illustrate how a variety of spatial data can be used to inform evidence-based decision-making in a wide range of fields. The *rspatialdata* website is a useful resource for individuals working with problems that require spatial data analysis and visualisation, such as estimating air pollution, quantifying disease burdens, predicting species occurrences, and evaluating and monitoring the UN Sustainable Development Goals.

An ongoing challenge in many disciplines that use spatial data is a lack of data in some locations and periods of time, as well as a lack of disaggregated data corresponding to age groups, genders and other factors. Spatial data are often aggregated at the scale of administrative units rather than locally relevant scales. These limitations make it difficult to compare processes over time and to evaluate outcomes for different population groups. While modeling techniques can be used to fill these gaps, ^85^^,^^86^ it is important to continue supporting countries to generate and access data that will help inform better decision-making globally.

We have chosen to write tutorials for spatial datasets that are important for decision-making in a wide range of fields such as health, climate, environment and ecology. While there may be different packages that do the same as the packages included in the website, *rspatialdata* tries to present the packages that are easiest to install and use, and includes other additional packages in the reference sections so users can explore additional functionalities and examples these packages provide. The website will be updated by including noteworthy packages to retrieve spatial data as they are discovered, and tutorials of existing packages will be updated if the code to use them changes or there are new notable functions to include. Also, in order to encourage the community to contribute, the website provides guidelines for contribution. The *rspatialdata* website is not comprehensive and it does not contain all available datasets. Nevertheless, it can provide a useful resource to get users started and a stimulus and location for others to contribute.

We expect the quantity and variety of spatial data provided by novel data streams such as satellite imagery, remote sensing, and GPS tracking to only increase in the future. The *rspatialdata* website will be regularly updated to meet the growing demands to access spatial data by the R community and to include new R packages and data sources as they are developed and released. By promoting the reuse and sharing of spatial data and spatial analyses, the *rspatialdata* website contributes to community-building and sharing of best practices on working with spatial data.

## Data availability

### Underlying data

Table 2 contains the databases included in the *rspatialdata* website.

**Table 2.** Databases included in the *rspatialdata* website.

Data	Database
Administrative boundaries	geoBoundaries
Population	WorldPop
OpenStreetMap	OpenStreetMap (OSM)
Elevation	AWS Terrain Tiles
Temperature	WorldClim
Rainfall	NASA-POWER Project
Humidity	NASA-POWER Project
Vegetation	Moderate Resolution Imaging Spectroradiometer (MODIS)
Land cover	Moderate Resolution Imaging Spectroradiometer (MODIS)
Air pollution	UK Department for Environment Food & Rural Affairs
Demographic and Health Surveys (DHS)	DHS Program
Malaria	Malaria Atlas Project (MAP)
Species Occurrence	Global Biodiversity Information Facility (GBIF)

## Software availability

Software available from: https://rspatialdata.github.io/.

Source code available from: https://github.com/rspatialdata/rspatialdata.github.io.

Archived source code at time of publication: https://doi.org/10.5281/zenodo.6779351. ^87^

License: MIT
