# Supplementary material for: rspatialdata: a collection of data sources and tutorials on downloading and visualising spatial data using R
Source: F1000Res. 2022 Jul 11;11:770. [Version 1] doi: 10.12688/f1000research.122764.1 (PMC9363973; doi:10.12688/f1000research.122764.1)
Supplement: Peer review history

# Peer review: Reviewer response for version 1

**DOI:** 10.5256/f1000research.134795.r144387

**Published:** 2022-08-09

**Authors:** Natalia da Silva

**Affiliations:** 1 Instituto de Estadística (IESTA), Universidad de la República, Montevideo, Uruguay

rspatialdata: a collection of data sources and tutors on downloading and visualizing spatial data using R.

 This paper presents a website that provides a collection of data sources and tutorials on downloading and visualizing spatial data using R. There are several R packages that simplify the access and reliability of spatial data but rspatialdata can help researchers to find and use spatial data in a simple way, tutorials are focused on read and manipulate data in a common analysis system promoting the data reuse and analyses.

**General comment:**

 Since you are presenting a webpage I think it will be good to include a general description of the webpage structure, describing each tabs, and at least an image with the Home tab describing the user interaction. In Methods before Table 1. Also, you should mention which tools you have used to design the webpage.

 It will be good to include a complete use case with all the visualizations as you have on the webpage, maybe on page 4 you can extend the temperature data example and show and comment on the figures, not just the R code.

**Minor comments:**

1. “R is a powerful language for statistical programming that incorporates a wide range of packages that can be used for data access, manipulation, analysis, and visualization.” This is a general statement not focused on spatial data and your references are specific and auto references to your work, you can include some general references or be specific to spatial data.
2. Page 3. It will be useful to include the webpage URL at least once https://rspatialdata.github.io for printed version. The same for the GitHub repo ( https://github.com/rspatialdata/rspatialdata.github.io).
3. Page 4 Table 1, second column contains the R packages, and since this is the first time you mentioned them in the paper I think you should include citation there. Not sure if there is any restrictions on including references in a table in this publishing platform.
4. Page 4 first paragraph, you should cite ggplot2.
5. Remove Table 2, it has the same info as Table 1.

Are the conclusions about the tool and its performance adequately supported by the findings presented in the article?

Yes

Is the rationale for developing the new software tool clearly explained?

Yes

Is the description of the software tool technically sound?

Yes

Are sufficient details of the code, methods and analysis (if applicable) provided to allow replication of the software development and its use by others?

Yes

Is sufficient information provided to allow interpretation of the expected output datasets and any results generated using the tool?

Yes

Reviewer Expertise:

I'm a Statistician interested in statistical computing, data visualization among others.

I confirm that I have read this submission and believe that I have an appropriate level of expertise to confirm that it is of an acceptable scientific standard.

# Peer review: Reviewer response for version 1

**DOI:** 10.5256/f1000research.134795.r144385

**Published:** 2022-07-29

**Authors:** Emmanuel Olamijuwon

**Affiliations:** 1 University of Southampton, Southhampton, UK

This paper describes a web platform (rspatialdata) that makes diverse population, health, climate and environmental data more Findable and Accessible. The website also provides instructions for accessing, exploring, and reusing (visualising) the datasets. As the authors note, high-resolution spatial data is crucial to tailoring management plans and service delivery to local situations. By promoting the reuse and sharing of spatial data and spatial analyses, the rspatialdata website contributes to community-building and sharing of best practices for working with spatial data.

 I agree with the authors that there's an increasing need for a central repository for information about spatial data sources and tutorials on their use. The tutorials are purposefully designed and well written in such a way that they will be easy to understand by anyone with basic r-programming experience. The rspatialdata website is also user-friendly, making it easy for anyone with an internet-enabled device to access them. In recognition of the ever-increasing variety of spatial data provided by novel data streams such as satellite imagery, remote sensing, and GPS tracking, the authors have also included a dedicated section for inviting community contributions, which is commendable.

 I have included a few minor comments below with the hopes that they would further strengthen the work.

- Table 1: I suggest changing Demographic and health survey to demographics and health (data focus).
- Table 2 is technically a repetition of Table 1. 
- Considering the article's peculiar focus on FAIR principles. It will be important to include a tutorial that demonstrates the interoperability of the datasets. That is, linking demographics and health data to Administrative boundaries or Humidity and/or Population data. I believe this would be a more substantial contribution of this article and website.
- Please include some spatial tutorials for the DHS module/page ( https://rspatialdata.github.io/dhs-data.html).

Are the conclusions about the tool and its performance adequately supported by the findings presented in the article?

Yes

Is the rationale for developing the new software tool clearly explained?

Yes

Is the description of the software tool technically sound?

Yes

Are sufficient details of the code, methods and analysis (if applicable) provided to allow replication of the software development and its use by others?

Yes

Is sufficient information provided to allow interpretation of the expected output datasets and any results generated using the tool?

Yes

Reviewer Expertise:

Demography and Social Statistics, Global Health; Africa

I confirm that I have read this submission and believe that I have an appropriate level of expertise to confirm that it is of an acceptable scientific standard.
